# Estimation of sodium and chloride storage in critically ill patients: a balance study

**DOI:** 10.1186/s13613-018-0442-2

**Published:** 2018-10-11

**Authors:** Lara Hessels, Annemieke Oude Lansink-Hartgring, Miriam Zeillemaker-Hoekstra, Maarten W. Nijsten

**Affiliations:** 1 Department of Critical Care, University of Groningen, University Medical Center Groningen, Hanzeplein 1, 9700 RB Groningen, The Netherlands; 2 Department of Anesthesiology, University of Groningen, University Medical Center Groningen, Groningen, The Netherlands

**Keywords:** Sodium, Chloride, Intensive care unit, Intracellular volume, Extracellular volume

## Abstract

**Background:**

Nonosmotic sodium storage has been reported in animals, healthy individuals and patients with hypertension, hyperaldosteronism and end-stage kidney disease. Sodium storage has not been studied in ICU patients, who frequently receive large amounts of sodium chloride-containing fluids. The objective of our study was to estimate sodium that cannot be accounted for by balance studies in critically ill patients. Chloride was also studied. We used multiple scenarios and assumptions for estimating sodium and chloride balances.

**Methods:**

We retrospectively analyzed patients admitted to the ICU after cardiothoracic surgery with complete fluid, sodium and chloride balance data for the first 4 days of ICU treatment. Balances were obtained from meticulously recorded data on intake and output. Missing extracellular osmotically active sodium (MES) was calculated by subtracting the expected change in plasma sodium from the observed change in plasma sodium derived from balance data. The same method was used to calculate missing chloride (MEC). To address considerable uncertainties on the estimated extracellular volume (ECV) and perspiration rate, various scenarios were used in which the size of the ECV and perspiration were varied.

**Results:**

A total of 38 patients with 152 consecutive ICU days were analyzed. In our default scenario, we could not account for 296 ± 35 mmol of MES in the first four ICU days. The range of observed MES in the five scenarios varied from 111 ± 27 to 566 ± 41 mmol (*P* < 0.001). A cumulative value of 243 ± 46 mmol was calculated for MEC in the default scenario. The range of cumulative MEC was between 62 ± 27 and 471 ± 56 mmol (*P* = 0.001 and *P* = 0.003). MES minus MEC varied from 1 ± 51 to 123 ± 33 mmol in the five scenarios.

**Conclusions:**

Our study suggests considerable disappearance of osmotically active sodium in critically ill patients and is the first to also suggest rather similar disappearance of chloride from the extracellular space. Various scenarios for insensible water loss and estimated size for the ECV resulted in considerable MES and MEC, although these estimates showed a large variation. The mechanisms and the tissue compartments responsible for this phenomenon require further investigation.

**Electronic supplementary material:**

The online version of this article (10.1186/s13613-018-0442-2) contains supplementary material, which is available to authorized users.

## Background

When long-term balance studies in humans demonstrated that sodium could accumulate without weight gain or hypernatremia, this challenged the generally accepted model on sodium homeostasis [1]. This model states that changes in sodium homeostasis can primarily be explained by a two-compartment model with an intracellular (ICV) and extracellular volume (ECV), where key ions are completely dissolved—i.e., osmotically active. An extra compartment that stores sodium nonosmotically without causing an expansion of the ECV has been proposed by Titze et al. [2]. In both animal and human studies, they found that sodium is stored nonosmotically in the skin [2, 3]. Nonosmotic sodium storage is presumably facilitated by large strongly negatively charged polymers such as glycosaminoglycans [4, 5]. The accumulation of chloride in the skin has been suggested in animal models [6, 7], but has not been as extensively studied as sodium storage.

Patients admitted to the intensive care unit (ICU) typically receive large amounts of sodium and chloride during their ICU treatment [8]. Both hypernatremia and hyperchloremia are a frequent complication in critically ill patients and are associated with adverse outcome [9–12]. The infusion of high amounts of chloride is also recognized as cause of hyperchloremic acidosis [11, 12]. Improved understanding of sodium chloride homeostasis in this patient group is therefore of utmost importance. To our knowledge, no studies have tried to measure missing sodium as evidence of stored sodium in ICU patients. Likewise, a potentially similar phenomenon for chloride has not been studied yet.

The objective of our study was therefore to estimate sodium and chloride that might ‘disappear’ in balance studies in ICU patients. Since random and systematic errors as well as different assumptions on the size of the ECV and perspiration strongly affect the calculated sodium or chloride deficit, five scenarios were tested in which the assumed sizes of the ECV or perspiration were varied.

## Methods

### Study design

This observational retrospective balance study involved all patients of ≥ 18 years admitted to a tertiary cardiothoracic ICU from October 2010 until December 2014 with a minimal ICU length of stay of 4 days.

### Data collection

Data that were collected and analyzed included basic demographics, reason of admission, acute physiology and chronic health evaluation (APACHE-IV) score for disease severity, acute kidney injury according to the KDIGO AKI criteria in the first 7 days and in-hospital mortality [13]. Fluid, sodium and chloride balances were derived from meticulously recorded input records (including enteral and parenteral feeding and administered fluids, including creep fluids such as solvent solutions) and output records, including daily 24-h urine collections. Our ICU did not have a full electronic patient database management system during the study period. Therefore, all data were derived from nursing and medical charts. All electrolyte concentrations, determined in blood or 24-h urine, were collected.

### Estimation of missing extracellular osmotically active sodium (MES) and chloride (MEC)

The most important components to determine electrolyte balances are detailed records of fluids, administered to or lost by the patient, including 24-h urine analyses. The detailed calculations used for determining water, sodium and chloride balances, including the estimation for (in)sensible perspiration, have been described earlier and are specified in detail in Additional file 1: Tables S1–S3 [8]. Insensible perspiration was calculated as:

- Insensible perspiration = 10 mL/kg/day + 2.5 mL/kg/day per degree centigrade above 37 °C (max body weight in equation 100 kg) (× 0.6 if intubated)(× 0.5 on admission day) [14].

For insensible perspiration, core temperature measured via the bladder catheter was used.

To estimate MES for each ICU patient, we compared the observed changes in estimated extracellular ΔNa_obs_ with the expected change (ΔNa_exp_).

Of every ICU calender day, last measured plasma sodium was compared with the last measured plasma sodium of the previous day. For electrolyte measurements, the direct ion-selective method was used. For the patients studied, we defined the ECV at 40% in our default model, since surgical patients receive a considerable fluid load perioperatively [15]. We corrected for different sizes of the ECV at the beginning of the day versus the end of day, due to infused fluids.

Only ECV_first_ that was calculated for admission day used measured body weight:

$${\text{ECV}}_{\text{first}} \, = \,0.4\, \times \,{\text{body}}\,{\text{weight}}\,\,\left( {\text{kg}} \right).$$

The extracellular volume at the end of the day (i.e., 23:59) was defined as:

$${\text{ECV}}_{23:59} \, = \,{\text{ECV}}_{\text{previous}} \, + \,{\text{fluid}}\,{\text{balance}}\,\,\left( {\text{L}} \right),$$

where ECV_previous_ is the ECV from 24 h earlier, or in the case it concerns the end of the first ICU day it relates to ECV_first_.

The extracellular volume at the beginning of the next day (i.e., 00:00) was defined as:

$${\text{ECV}}_{00:00} = {\text{ECV}}_{{{\text{last}}\,{\text{of}}\,{\text{the}}\,{\text{previous}}\,{\text{day}}}} \,\,\left( {\text{L}} \right).$$

The expected change in total amount of sodium in the ECV over a calender day was defined as:

$$\Delta {\text{Na}}_{ \exp }^{ + } \, = \,\left[ {{\text{Na}}^{ + } } \right]_{\text{last}} \, \times \,{\text{ECV}}_{\text{last}} \, - \,\left[ {{\text{Na}}^{ + } } \right]_{\text{previous}} \, \times \,{\text{ECV}}_{\text{previous}} \,\,\left( {\text{mmol}} \right).$$

The observed change in total extracellular sodium on the basis of administrated and excreted sodium was thereafter defined as:

$$\Delta {\text{Na}}_{\text{obs}}^{ + } \, = \,{\text{sodium}}\,{\text{balance}}\, = \,{\text{Na}}_{\text{in}} \,{-}\,{\text{Na}}_{\text{out}} \,\,\left( {\text{mmol}} \right).$$

The missing extracellular osmotically active sodium that apparently ‘disappeared’ from the ECV was defined as:

$${\text{MES}}\, = \,\Delta {\text{Na}}_{\text{obs}} \,{-}\,\Delta {\text{Na}}_{ \exp } \,\,\left( {\text{mmol}} \right).$$

For chloride, the same method as described above was used to calculate MEC, where instead of sodium, chloride should be read.

As a sensitivity analysis to test the robustness of our results, we tested 2 × 2 additional more extreme scenarios with respect to our assumptions on the ECV and perspiration. Where the default model assumed an ECV of 40% of the body weight and an insensible perspiration of 10 mL/kg/day, we tested both an extracellular compartment of 20% of body weight [16] and 60% of body weight [17]. In order to encapsulate the wide uncertainty in estimating actual perspiration, we also tested both lower and upper published extremes in perspiration rate of 5 mL/kg/day plus 2.5 mL/kg/day per degree centigrade above 37 °C versus and a perspiration rate of 20 mL/kg/day plus 2.5 mL/kg/day per degree centigrade above 37 °C.

To assess the differences in ECV between males and females, we performed a sub-analysis. In this analysis, an ECV of 40% of the body weight was assumed for males and an ECV of 30% was assumed for females.

### Statistical analyses

Means are given ± SE, medians with interquartile range, unless otherwise indicated. MES and MEC were compared with a Student’s *t* test. A two-sided *P* < 0.05 was considered significant. Cumulative calculations took account of increases in cumulative errors with the Pythagorean theory of error propagation. Balance calculations and statistical analysis were performed with SPSS 23.0 (IBM, Chicago, IL).

## Results

A total of 38 patients with 152 consecutive ICU days were included. Their baseline characteristics are given in Table 1.

**Table 1:** Patients characteristics

	*n* = 38
Age (years)	66 (13)
Sex, male	28 (74%)
Reason of admission	
Cardiothoracic surgery	31 (82%)
Trauma	1 (3%)
Vascular surgery	1 (3%)
*Miscellaneous*	5 (13%)
LOS ICU (days)	7.4 (4.8–13.7)
Patients on diuretics	25 (66%)
APACHE-IV	60 (44-71)
Hospital mortality	4 (11%)
AKI	11 (29%)
Stage 1	6 (55%)
Stage 2	3 (27%)
Stage 3	2 (18%)

Data are depicted as mean (SD), *n* (%) or median (interquartile range) as appropriate

*APACHE* Acute Physiology and Chronic Health Evaluation

The included patients received large amounts of fluids (13.6 ± 0.6 L), sodium (1441 ± 75 mmol) and chloride (1377 ± 76 mmol) in the 4-day period, resulting in a cumulative fluid balance of 3.9 ± 0.6 L, a sodium balance of 822 ± 76 mmol and a chloride balance of 556 ± 82 mmol. Both the mean plasma sodium and chloride concentrations did not significantly change during the first four ICU days (Table 2).

**Table 2:** Cumulative data on fluid and electrolyte administration

	Day 1	Day 2	Day 3	Day 4	P*
*Intake*					
Fluid (L)	3.6 ± 0.4	7.9 ± 0.5	11.0 ± 0.5	13.6 ± 0.6	
Sodium (mmol)	460 ± 52	935 ± 67	1235 ± 72	1441 ± 75	
Chloride^a^ (mmol)	420 ± 53	872 ± 68	1162 ± 79	1377 ± 76	
*Output*					
Fluid (L)	1.6 ± 0.1	3.9 ± 0.2	6.5 ± 0.3	8.9 ± 0.3	
Sodium (mmol)	139 ± 14	293 ± 23	472 ± 30	626 ± 35	
Chloride^a^ (mmol)	117 ± 17	255 ± 28	418 ± 43	574 ± 47	
*Balance*					
Fluid (L)	1.9 ± 0.3	3.7 ± 0.5	4.0 ± 0.5	3.9 ± 0.6	
Sodium (mmol)	321 ± 47	642 ± 62	769 ± 72	822 ± 76	
Chloride^a^ (mmol)	274 ± 51	498 ± 66	535 ± 82	556 ± 82	
Plasma sodium (mmol/L)	137.1 ± 0.5	136.6 ± 0.5	136.2 ± 0.6	136.9 ± 0.5	0.741
Plasma chloride^a^ (mmol/L)	109.8 ± 0.9	109.3 ± 0.8	108.2 ± 0.9	108.0 ± 0.9	0.107

Data are depicted as mean ± SE

* Difference between day 1 and day 4

^a^Chloride data were available for 27, 27, 24 and 28 patients, respectively, on day 1 to day 4

### Missing extracellular osmotically active sodium and chloride

**Fig. 1:**
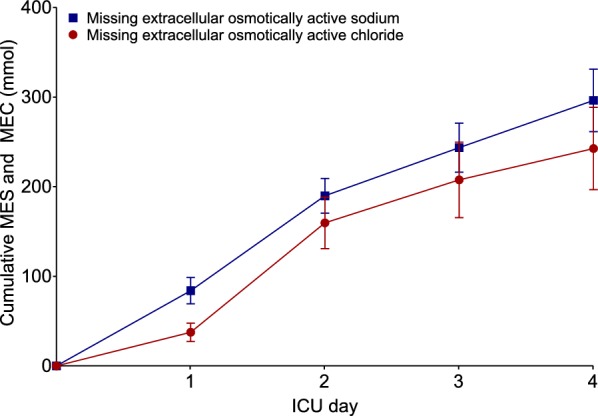
Time course of estimated cumulative MES and MEC for the first four ICU days. Values are depicted as mean ± SE. The first values reflect levels at ICU admission, when storage was assumed defined as zero. The values at the subsequent time points reflect levels at the end (i.e., midnight) of each ICU day. As can be seen under normal and stable circulating electrolyte levels (Table 2), a significant amount of sodium (MES) and chloride (MEC) ‘disappears’ from the balances over the first four ICU days

Based on our calculations, for sodium a MES of 74 ± 15 mmol per day was observed. This resulted in a cumulative MES of 296 ± 35 mmol during four ICU days (Fig. 1). For chloride, a MEC of 61 ± 23 mmol per day was seen with a cumulative MEC of 243 ± 46 mmol over the first four ICU days (Fig. 1).

We also calculated the difference between MES and MEC. The cumulative difference was 56 ± 40 mmol over the first four ICU days.

### Scenarios

**Fig. 2:**
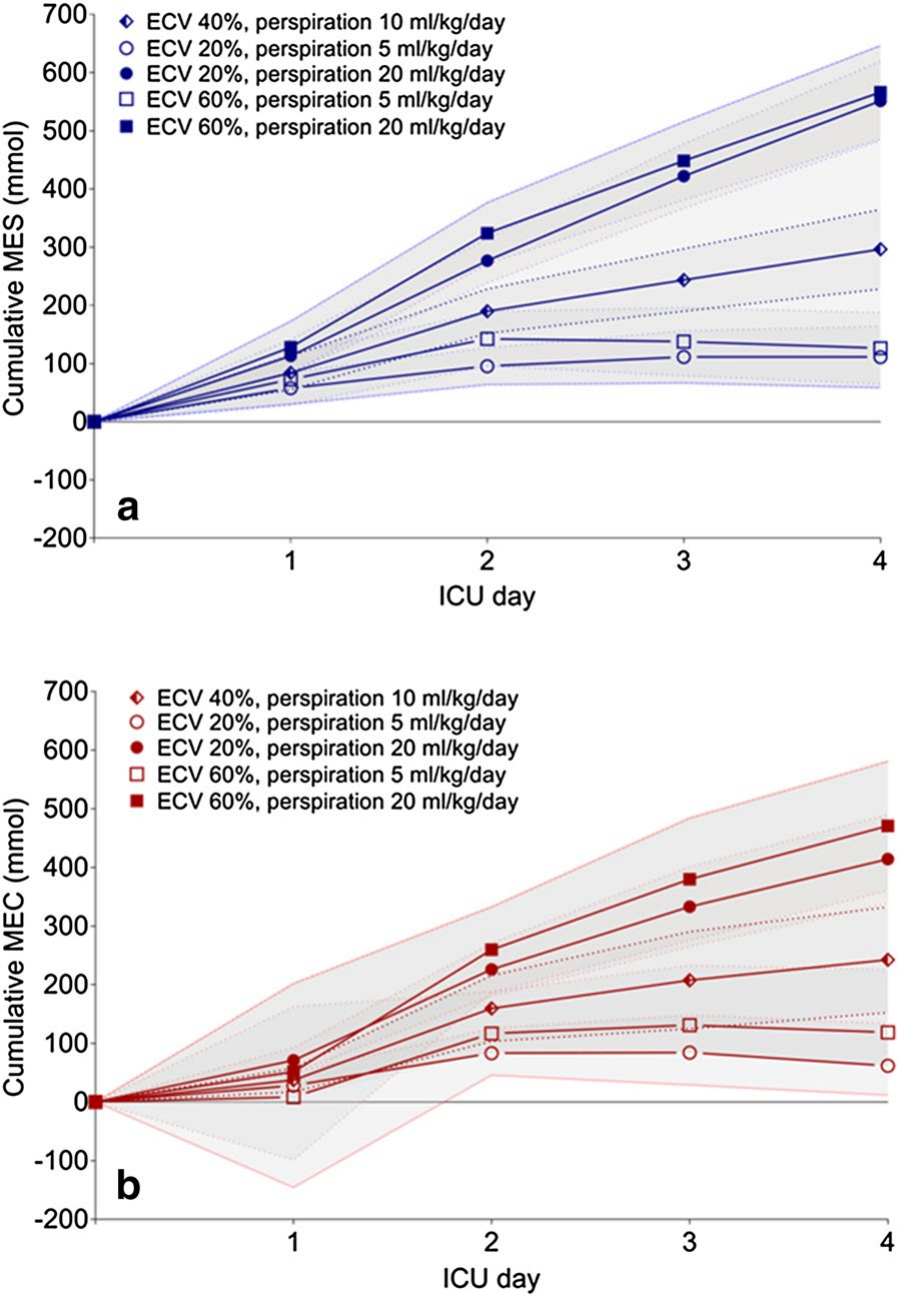
Scenarios for both estimated cumulative MES and MEC. Values are depicted as mean ± 95% CI. The 95% CI is represented by the dotted lines. The first values reflect levels at ICU admission, when storage was assumed to be zero. In all scenarios, there were considerable MES and MEC after 4 days of ICU admission. **a** With stable sodium levels, MES is mostly influenced by altering the insensible perspiration. **b** MEC showed a similar pattern as MES, but was slightly more affected by the changes in the extracellular compartment than MES

**Fig. 3:**
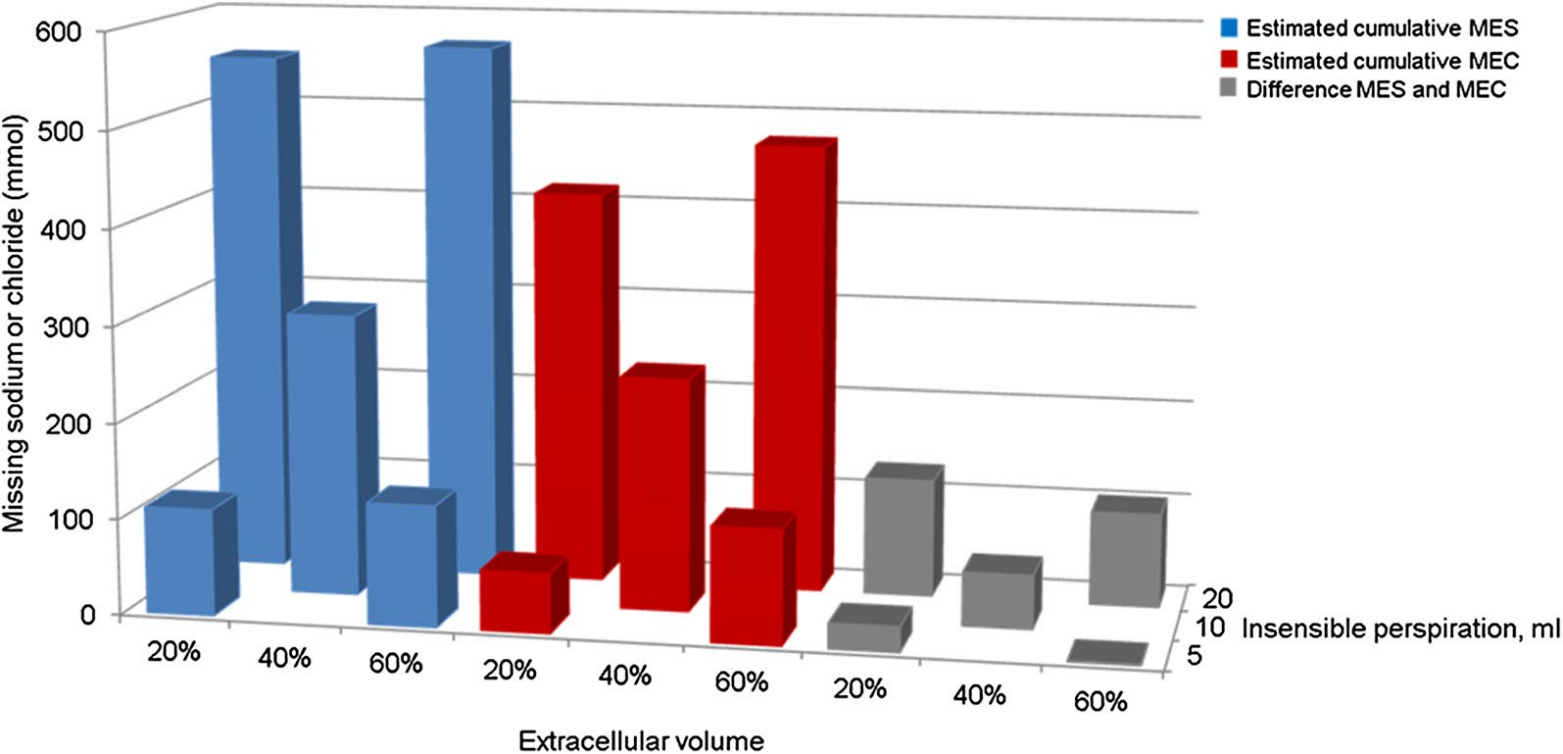
Estimated cumulative MES and MEC according to different scenarios. Values are depicted as means. The calculated MES (blue), MEC (red) and their difference (light gray) on ICU day 4 according to the scenarios with different assumptions on perspiration and the size of the extracellular volume

In the four scenarios in addition to the default scenario, we changed the assumed ECV and assumed perspiration and assessed their impact on MES and MEC (Figs. 2 and 3).

When perspiration was increased to 20 mL/kg/day, MES doubled for both the 20% and 60% ECV scenario (551 ± 35 mmol and 566 ± 41 mmol, respectively, both *P* < 0.001, Fig. 2a). After decreasing perspiration to 5 mL/kg/day, in both the ECV of 20% and 60% scenario, the amount of sodium we could not account for decreased more than 2.5 times the initial calculated MES (111 ± 27 mmol and 126 ± 31 mmol, respectively, both *P* < 0.001).

When these four scenarios were repeated for the calculations of MEC, similar results were obtained. When perspiration was increased to 20 mL/kg/day, for both the 20% and 60% ECV scenario we observed a similar increase in MEC from 243 ± 46 mmol to 414 ± 39 mmol (*P* = 0.006) to and 471 ± 56 mmol (*P* = 0.003, Fig. 2b). After decreasing perspiration to 5 mL/kg/day, MEC decreased. A MEC of 62 ± 27 mmol was observed when ECV was set at 20% in the low perspiration scenario (*P* = 0.001). However, when ECV was defined as 60%, MEC did not significantly change (243 ± 46 mmol vs. 119 ± 55 mmol, respectively, *P* = 0.09).

For all five depicted scenarios, the difference between MES and MEC was also calculated (Fig. 3) to identify potential structural differences between sodium and chloride disappearance. These differences varied from 1 ± 51 to 123 ± 33 mmol, indicating that MES and MEC were of the same order of magnitude.

The sub-analysis to assess sexual differences in ECV is found in Additional file 1: Fig. S1.

## Discussion

This is the first balance study that aimed to estimate missing extracellular sodium (MES) in ICU patients and missing extracellular chloride (MEC) in any patient group. Although we found considerable variations in estimated MES and MEC according to the various scenarios, the results suggest a considerable MES and a somewhat lower MEC (Fig. 3).

To calculate MES and MEC, we used one default and four more extreme scenarios, which we believe cover the scope of published sizes of the ECV and rates of perspiration. An ECV of 20% of body weight is a conservative choice in patients arriving at the ICU after major surgery [16], while 60% is an extreme estimate [17]. Regarding perspiration, defining the extremes was more difficult, but nearly all sources assume a perspiration ≥ 400 ml/day for both the skin and the respiratory tract without fever [14]. Our estimate of 5 mL/kg/day probably is thus the lower limit, while 20 mL/kg/day is a large estimate. We believe that the true value of both MES and MEC should be somewhere in between the four more extreme scenarios as depicted in Fig. 3. MES and MEC were mainly influenced by perspiration and MEC also somewhat by the ECV. This underscores that both the size of the ECV and insensible perspiration are important determinants in the estimated size of MES and MEC. In sex-specific models (Additional file 1: Fig. S1), males showed slightly higher MES and MEC compared with females.

More sodium and chloride disappeared from the balances during the first two days of ICU admission than in the subsequent days (Fig. 1). Resuscitation fluids, often high in sodium and chloride content, are frequently administered during surgery and in the early postoperative period. Whereas the recommended limits for dietary sodium intake are 2.3 g/day [18], our patients received an average of 8.3 g (i.e., 360 mmol) sodium per day, with positive sodium balances but stable sodium concentrations. This resulted in a MES of 296 mmol after four ICU days. When this MES is expressed in terms of NaCl 0.9% infusion, 1.9 L of this fluids sodium went missing in our patients.

Nonosmotic sodium storage has been studied in several non-critically ill patient groups. In healthy individuals, it has recently been observed that half of an acute intravenous hypertonic saline load of 201 mmol appears to be briefly stored nonosmotically [19], possibly in interaction with the endothelial glycocalyx. Sodium storage has been reported to increase with advancing age, to be greater in men and patients with hypertension, hyperaldosteronism, end-stage kidney disease and infection [20–22]. Tissue sodium levels are variable and may be altered by dialysis and diuretic treatment [23, 24]. However, the precise clinical significance of nonosmotic sodium storage has not been defined yet. The existence of nonosmotic sodium storage has not been examined in critically ill patients. Nonosmotic sodium storage could also be relevant in ICU-acquired hypernatremia (IAH) [25] and could explain the relatively long duration of IAH once it develops, although sodium balances were not performed in this study. It is believed that the electrical binding capacity of various tissues for sodium is altered during inflammation [26], which may interact with the development of IAH in critically ill patients. Irrespective of a potential relation between IAH and sodium storage, a strategy in which infusion fluids with lower sodium chloride content are used to reduce IAH is probably desirable [27]. We reported earlier [10] that changes in bulk intravenous fluid constitution paralleled changes in the incidence of ICU-acquired hypernatremia. Recently, it was elegantly shown that maintenance fluid therapy constitutes a higher sodium, chloride and water burden than acute resuscitation fluid administration [28].

With regard to chloride, which also disappeared in our balance calculations, both sodium and chloride storage may affect changes in blood pressure [6, 29].

As we cannot explain MES and MEC by the conventional two-compartment model where sodium is extracellular and potassium intracellular, a specific storage compartment may be the buffer of these sodium and chloride loads. The key alternative to nonosmotic storage is loss of sodium and chloride to the ICV. This effect has been demonstrated by healthy persons who sustained muscular injury [30]. Critical illness is often accompanied with critical illness myopathy, and loss of sodium and chloride to the ICV might then also be conceivable [31]. As we reported in an earlier study [8], our patients displayed a *negative* potassium balance of 101 mmol, which is another argument for possible intracellular uptake of sodium in exchange for potassium release. Moreover, we also observed a negative electrolyte-free water (EFW) balance in these patients. Together, this suggests that no ICV expansion occurred [8]. Therefore, we assumed that all fluids administered (including EFW) remained in the ECV. However, if part of the EFW would enter the ICV, this would result in lower increases and thus even higher MES and MEC estimates.

The presence of nonosmotic storage could be verified through direct tissue analysis or via specialized MRI [2, 3, 20]. Sodium changes in the tissues of ICU patients resulting from MES could be imaged via ^23^Na MRI. To our knowledge, ^35^Cl MRI has not yet been used to study MEC, but it is a promising and intriguing technique to identify the anatomical spaces where salt is stored [32, 33]. Importantly, this technique should be able to differentiate between the two main explanations for missing sodium and chloride: nonosmotic storage or intracellular uptake.

Our study has a number of limitations. Due to its retrospective design, we could not control for many variations in standard care. We had to make several assumptions, as, for example, for the insensible perspiration or the size of the ECV. However, we believe that the extreme scenarios on perspiration and ECV in our sensitivity analyses covered all realistic scenarios. We did not account for fecal losses, as we could not retrieve this information. Since we observed early postoperative patients, fecal production was absent or very low and, moreover, loss of sodium and chloride through the gut is usually very limited [34]. We did not measure weight changes as this is not routine procedure at our unit. Daily weight measurements could be added in the future to further validate our results.

On the relatively short term, *differences* in body weight measured in kg as measured in ICU patients will be less accurate than fluid balances measured in mL. Therefore, we only used initial recorded weight to estimate the ECV. Fluid balances in critically ill patients often have a poor correlation with changes in body weight [35, 36]. Especially cumulative fluid balance is prone to errors, as measuring errors get cumulated [36], which we accounted for in our error estimates. It must be noted that body weight measurement also has multiple possible errors, which could be the explanation of the lack of association between fluid balance and differences in body weight [36]. However, we believe that due to the short time this study covers and the meticulous recalculation of the fluid balance, including gastric retention, drain fluids and insensible perspiration, we have minimized errors as far as realistically possible.

Insensible perspiration remains very challenging to measure. As MES and MEC were most influenced by insensible perspiration, the lack of direct measurement of perspiration is an important limitation of our study. We tried, however, to maximize the chance to include the true value as much as possible with our five different scenarios. Direct measurement of (in)sensible perspiration would make estimated of MES and MEC more accurate (Fig. 3). Unfortunately, we are not aware of reliable tools to measure (in)sensible perspiration.

In this first observational balance study, we selected our patients based on complete balance data, which could have induced selection bias. The Androque-Madias [37] and Nguyen-Kurtz [38] formulas are frequently used when estimating the plasma sodium level after a saline infusion in dysnatremic ICU patients [17, 19]. However, we choose not to use these formulas in our study, as they do not account for excretion of sodium or chloride or they use empirically derived constants which were not suitable for using in our model. However, predictions on the size of the ECV from both formulas fall within the four scenarios.

In conclusion, our detailed sodium and chloride balances in ICU patients after cardiothoracic surgery suggest a loss of osmotically active sodium and chloride from the ECV. The estimates depend considerably on the scenarios used. Whether these ions are nonosmotically stored or transferred to the intracellular space needs further study.

## Additional file

**Additional file 1.** Detailed information on constants and calculations and sex-specific model for MES and MEC.

## Abbreviations

AKI: acute kidney injury
APACHE-IV: Acute Physiology and Chronic Health Evaluation—IV
ECV: extracellular volume
ICU: intensive care unit
ICV: intracellular volume
MEC: missing extracellular osmotically active chloride
MES: missing extracellular osmotically active sodium
MRI: magnetic resonance imaging
[Na^+^]_first_: plasma sodium concentration on day of measurement
[Na^+^]_last_: last plasma sodium concentration previous day
ΔNa_obs_: observed change in estimated extracellular sodium
ΔNa_exp_: expected change in estimated extracellular sodium
