# Supplementary material for: Estimation of sodium and chloride storage in critically ill patients: a balance study
Source: Ann Intensive Care. 2018 Oct 11;8:97. doi: 10.1186/s13613-018-0442-2 (PMC6179979; doi:10.1186/s13613-018-0442-2)
Supplement: Supplementary file 1 — Additional file 1. Detailed information on constants and calculations and sex-specific model for MES and MEC. [file 13613_2018_442_MOESM1_ESM.docx]

**Additional file**

To: **Estimation of sodium and chloride storage in critically ill patients: a balance study**By Hessels et al.

**Content**

Tables

**S1.** Constants and calculations Pg. 2

**S2.** Electrolyte content of infusion fluids Pg. 3

**S3.** Solutions used to dissolve frequently used medication. Pg. 4

All tables are compatible with : *Hessels L et al. Postoperative fluid retention after heart surgery is accompanied by a strongly positive sodium balance and a negative potassium balance. Phys Rep. 2016;4:e12807*

Figures

**S1.** Time course of estimated cumulative MES and MEC for the first four ICU Pg. 5

days in males and females

**Table S1. Constants and calculations**

| *Calculations* |
| --- |
| *Intake of water, sodium and chloride* |
| Intake = resuscitation fluids + solvent fluids + parenteral feeding + blood products + fluids to keep an open venous line + water (oral) (oral) |
| *Output of water, sodium and chloride* |
| Output = gastric retention + drain production + insensible perspiration + diuresis (24h urines) |
| *Balance of water, sodium and chloride* Balance= intake – output |
| Balance = intake – output |
| Insensible perspiration: 10 ml/kg/day |
| + 2.5ml/kg/day per degree centigrade above 37^o^C |
| (max body weight in equation: 100 kg) |
| * 0.6 if intubated |
| * 0.5 on admission day |
| Gastric retention: Volume * [electrolyte]_enteral/parenteral feeding_ (see Table 2) |
| Drain fluid loss: Volume * mean blood [electrolyte] |
| Temperature Mean body temperature of the day (mean of |
| temperature at 6h and 18h) |
| *Blood (mmol/L)* |
| Mean blood sodium 132 |
| Mean blood chloride: 108  EFW IFluid volume – ((Na^+^ mmol + K^+^ mmol)/140) |
|  |
| *Scenarios* |
| Default ECV_last_ = 0.4 x body weight (kg) + fluid balance (L) |
| Insensible perspiration: 10 ml/kg/day |
| A ECV_last_ = 0.2 x body weight (kg) + fluid balance (L) |
| Insensible perspiration: 5 ml/kg/day |
| B ECV_last_ = 0.6 x body weight (kg) + fluid balance (L) |
| Insensible perspiration: 5 ml/kg/day |
| C ECV_last_ = 0.2 x body weight (kg) + fluid balance (L) |
| Insensible perspiration: 20 ml/kg/day |
| D ECV_last_ = 0.6 x body weight (kg) + fluid balance (L) |
| Insensible perspiration: 20 ml/kg/day |

**Table S2. Electrolyte content of infusion fluids**

|  | **[Cl^-^](mmol/L)** | **[Na^+^] (mmol/L)** |
| --- | --- | --- |
| *Resuscitation fluids* | | |
| Glucose 2.5%/NaCl 0.45% | 77 | 77 |
| Glucose 5% | 0 | 0 |
| Glucose 50% | 0 | 0 |
| Lactated Ringers | 111 | 134 |
| NaCl 0.9% | 154 | 154 |
| NaCl 5% | 856 | 856 |
| Sterofundin® | 127 | 145 |
| Voluven® | 154 | 154 |
| *Parenteral/enteral feeding* | | |
| Nutridrink® | 40.67 | 24.54 |
| Nutrison concentrated® | 22.57 | 43.5 |
| Nutrison multifibre® | 35.27 | 43.5 |
| Nutrison protein plus® | 22.57 | 48.26 |
| Peptisorb® | 35.27 | 43.5 |
| TPN | 45 | 35 |
| *Blood products* | | |
| Albumin 20% | 100 | 100 |
| Cell saver blood | 100 | 140 |
| Cirrestor blood | 0 | 140 |
| FFP | 80 | 172 |
| Fibrinogen | 0 | 71 |
| Platelet concentrate | 70 | 120 |
| RBC | 80 | 126 |
|  |  |  |

**Table S3. Solutions used to dissolve frequently used medication**

| **Type of medication** | **Dissolved in infusion fluid*** |
| --- | --- |
| Adrenaline 10 mg/50 ml | NaCl 0.9% |
| Amiodarone 600mg/50 ml | Glucose 5% |
| Clonidine 600 ug/50 ml | NaCl 0.9% |
| Dexmedetomidine | Glucose 5% |
| Dobutamine 250mg/50ml | NaCl 0.9% |
| Dopamine 200mg/50 ml | NaCl 0.9% |
| Fluxocacillin | NaCl 0.9% ( [Na^+^]_end_ =418 mmol/L) |
| Furosemide 80 mg/50 ml | NaCl 0.9% |
| Ganciclovir | NaCl 0.9% |
| Heparin 20,000 IU/50 ml | NaCl 0.9% |
| Hydrocortisone 200 mg/50 ml | NaCl 0.9% |
| Insulin 50 IU/50 ml | NaCl 0.9% |
| Labetalol 250 mg/50 ml | None |
| Levosimendan | Glucose 5% |
| Magnesium sulfate | NaCl 0.9% |
| Midazolam 100mg/50 ml | NaCl 0.9% |
| Milrinone 10 mg/50 ml | NaCl 0.9% |
| Morphine 100mg/50 ml | NaCl 0.9% |
| Mycophenolate mofetil | Glucose 5% |
| Naloxone | NaCl 0.9% |
| Nicardipin 10 mg/50 ml | NaCl 0.9% |
| Nitroglycerin 10 mg/50 ml | NaCl 0.9% |
| Noradrenaline 10 mg/50ml | Glucose 5% |
| Phenylephrine | NaCl 0.9% |
| Piperacillin/Tazobactam (4/500) | Water ( [Na^+^]_end_ =196 mmol/L) |
| Propofol 2% | None |
| Protamine | NaCl 0.9% |
| Sodium phosphate | NaCl 0.9% |
| Tacrolimus 2mg/50 ml | NaCl 0.9% |
| Tranexaminic acid | NaCl 0.9% |
| Vasopressin 40 U/40 ml | NaCl 0.9% |

Legend to table ST3. *****Infusion fluids according to our institutions protocol at the time of the study. Since then several dissolving fluids have been changed into glucose 5%.


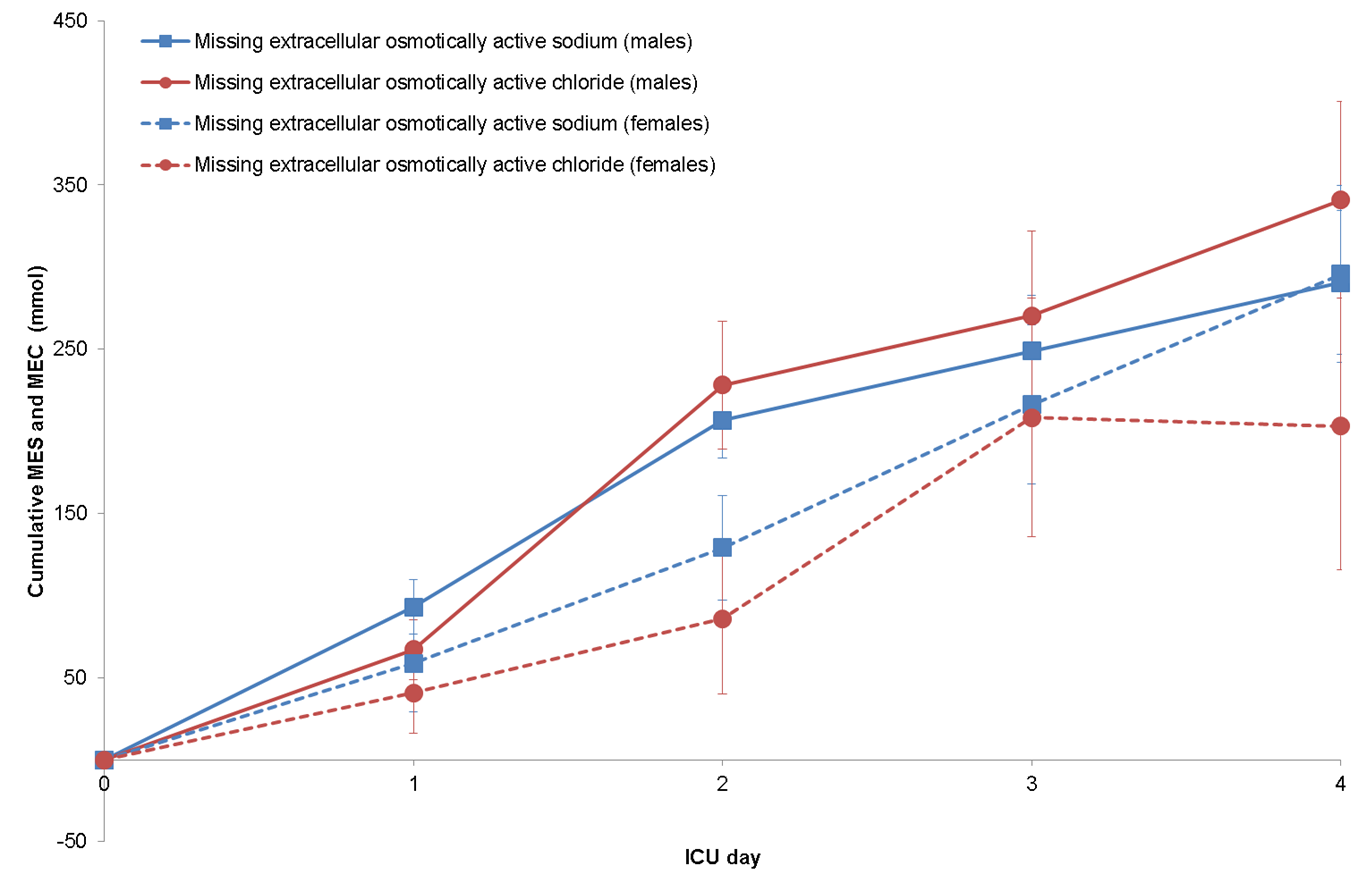


**Figure S1. Time course of estimated cumulative MES and MEC for the first four ICU days in males and females**

**Legend to Figure SF1.** Values are depicted as mean±SE. The first values reflect levels at ICU-admission, when storage was assumed defined as zero. The values at the subsequent time points reflect levels at the end (i.e. midnight) of each ICU day. As can be seen under normal and stable circulating electrolyte levels (Table 2), a significant amount of sodium (MES) and chloride (MEC) ‘disappears’ from the balances over the first four ICU-days. ECV has been defined as 40% of bodyweight for males and 30% of bodyweight for females. At day 4 the differences between males and females were not significant (MES: P= 0.95 , MEC P= 0.23).
